# Supplementary material for: Black soil layer thickness prediction and soil erosion risk assessment in a small watershed in Northeast China
Source: PLoS One. 2025 Jun 9;20(6):e0324368. doi: 10.1371/journal.pone.0324368 (PMC12148179; doi:10.1371/journal.pone.0324368)
Supplement: Attachment — Submitted filename: Response to Reviewers.docx [file pone.0324368.s002.docx]

1. When submitting your revision, we need you to address these additional requirements.

Please ensure that your manuscript meets PLOS ONE's style requirements, including those for file naming. The PLOS ONE style templates can be found at

https://journals.plos.org/plosone/s/file?id=wjVg/PLOSOne_formatting_sample_main_body.pdf and

<https://journals.plos.org/plosone/s/file?id=ba62/PLOSOne_formatting_sample_title_authors_affiliations.pdf>

The format of the article has been corrected according to the formatting requirements of your journal.

2. We note that the grant information you provided in the ‘Funding Information’ and ‘Financial Disclosure’ sections do not match.

When you resubmit, please ensure that you provide the correct grant numbers for the awards you received for your study in the ‘Funding Information’ section.

Answer：Revisions have been made to ‘Funding Information’ and ‘Financial Disclosure’.

3. In the online submission form you indicate that your data is not available for proprietary reasons and have provided a contact point for accessing this data. Please note that your current contact point is a co-author on this manuscript. According to our Data Policy, the contact point must not be an author on the manuscript and must be an institutional contact, ideally not an individual. Please revise your data statement to a non-author institutional point of contact, such as a data access or ethics committee, and send this to us via return email. Please also include contact information for the third party organization, and please include the full citation of where the data can be found.

Answer: The contact person for data is the Key Laboratory of Blackland Evolution and Ecological Effects, Shenyang Geological Survey Centre, China Geological Survey. Tel: +86-024-86002998. Email: cgssyzx_data@yeah.net

4. PLOS requires an ORCID iD for the corresponding author in Editorial Manager on papers submitted after December 6th, 2016. Please ensure that you have an ORCID iD and that it is validated in Editorial Manager. To do this, go to ‘Update my Information’ (in the upper left-hand corner of the main menu), and click on the Fetch/Validate link next to the ORCID field. This will take you to the ORCID site and allow you to create a new iD or authenticate a pre-existing iD in Editorial Manager.

Answer：Modified in the system

5. We note that Figure 1 in your submission contain [map/satellite] images which may be copyrighted. All PLOS content is published under the Creative Commons Attribution License (CC BY 4.0), which means that the manuscript, images, and Supporting Information files will be freely available online, and any third party is permitted to access, download, copy, distribute, and use these materials in any way, even commercially, with proper attribution. For these reasons, we cannot publish previously copyrighted maps or satellite images created using proprietary data, such as Google software (Google Maps, Street View, and Earth). For more information, see our copyright guidelines: <http://journals.plos.org/plosone/s/licenses-and-copyright>.

Answer：Figure 1 contains [map/satellite] images from NASA platforms. This data is free for use, modification, and distribution, including academic publication, in accordance with NASA's open data services and software strategy. Data sources have been cited in the text . [Open Data, Services, and Software Policies | NASA Earthdata](https://www.earthdata.nasa.gov/engage/open-data-services-software-policies)

6. Additional Editor Comments:

Two anonymous reviewers have reviewed the manuscript. The manuscript requires substantial revisions to address the issues in the present form. The Random Forest method's validation on a smaller range of observed values (30–60) as opposed to the entire range (10–130) covered by the kriging methods is one of the main concerns brought up by the reviewers. This disparity calls into question both the stated high R2 and the validity of the Random Forest results. To guarantee reliable comparisons, this approach must be tested across the entire spectrum. Furthermore, the wording "using raster calculations" in Table 1's description of precipitation data interpolation is imprecise. The paper should discuss the drawbacks of integrating coarse-resolution data with pixel sizes of 30 m and make clear how interpolation techniques affect the results. A comprehensive language evaluation is advised since the language has to be improved. The overall impact is limited by the study's unclear combination of soil erosion risk assessment and mapping the thickness of the black soil layer. A more unified process can result from including soil erosion as a variable in thickness mapping. The title has to be clarified, the units in the figures need to be formatted correctly, the novelty of this work needs to be better discussed with references to other research, and the sample approach needs to be clearly defined.

The Random Forest method's validation on a smaller range of observed values (30–60) as opposed to the entire range (10–130) covered by the kriging methods is one of the main concerns brought up by the reviewers. This disparity calls into question both the stated high R^2^ and the validity of the Random Forest results. To guarantee reliable comparisons, this approach must be tested across the entire spectrum.

Answer：In the previous study due to the non-normality of the black soil layer thickness data, the random forest model was over-sampled, resulting in an exaggerated R^2^ (0.79). This time, overfitting was prevented by limiting the depth of the random forest decision tree, and an R^2^ of 0.57 was obtained.

Furthermore, the wording "using raster calculations" in Table 1's description of precipitation data interpolation is imprecise. The paper should discuss the drawbacks of integrating coarse-resolution data with pixel sizes of 30 m and make clear how interpolation techniques affect the results.

Answer：The monthly precipitation data in Table 1 were rastered to obtain annual average precipitation data. The precipitation and dem data were then resampled to 30m resolution. Considering that the data are continuous and resampled using the averaging method, although the process loses some data precision, the margin of error is known to be sufficiently small so that no further errors are reported.

The overall impact is limited by the study's unclear combination of soil erosion risk assessment and mapping the thickness of the black soil layer. A more unified process can result from including soil erosion as a variable in thickness mapping.

Answer： Soil thickness, as an intrinsic physical property, plays a pivotal role in determining soil stability, water retention capacity, and erosion resistance. Particularly in regions characterized by intense precipitation or steep topographic gradients, thicker soil profiles demonstrate enhanced resistance to erosional processes, effectively mitigating the impact of external erosive forces. Conventional soil erosion assessment predominantly relies on a unidimensional approach utilizing the soil erosion modulus, which solely quantifies erosion intensity per unit time. However, the erosion modulus and soil thickness were combined to create an integrated two-dimensional assessment model of “erosion intensity - soil resistance”. enabling more accurate soil erosion risk assessment. This innovative model classifies erosion risk into four distinct categories: (1) high-risk areas characterized by high erosion modulus coupled with thin soil layers; (2) medium-risk areas exhibiting high erosion modulus with thick soil layers; (3) low-risk areas featuring low erosion modulus and thin soil layers; and (4) risk-free areas demonstrating both low erosion modulus and substantial soil thickness.

When the random forest model encounters highly correlated features, it may "overfit" certain variables, leading to a decrease in regression accuracy. The erosion modulus calculated by RUSLE has a strong linear or nonlinear correlation with input variables of the random forest model (such as slope, vegetation index, elevation, precipitation, etc.). Therefore, when it is included in the random forest model, it may lead to information redundancy, which can affect the model's performance. As a result, the RUSLE results were not used as input variables for predicting soil thickness.

The title has to be clarified, the units in the figures need to be formatted correctly.

Answer：The title has been revised in accordance with the review comments and the formatting of the units in the text has been revised.

The novelty of this work needs to be better discussed with references to other research.

Answer：Recent research advances have been added and compared to the present study.

The sample approach needs to be clearly defined

Answer：Route setting principles and black soil layer definitions have been added so that the article can be easily understood.

Reviewer #1: Thank you for the opportunity to review this research. The topic is important and the research approach is good, but I have some concerns about the execution and communication. I hope this can be revised to reach a broader audience though probably not in this journal.
My main concern pertains to Figure 4: All of the kriging methods show data points with observed values ranging from 10 to 130, matching the range of values reported in the paper. However, the Random Forest method only shows observed values ranging from about 30 to 60, and the linear regression is based on this small range. I don't think the overall results and much larger R^2^ can be reliable if the Random Forest method is not tested against all of the observed values.
Answer: Bias in the black soil thickness data in the previous model resulted in an exaggerated R^2^ (0.79). The R^2^ was now changed to 0.57 by limiting the depth of the decision tree to prevent overfitting.

A secondary concern pertains to the methods used to interpolate the precipitation data listed in Table 1. The methods section should say more than "using raster calculations" (as on lines 139 and 142) and should discuss the relative accuracy or error inherent in combining data from much coarser resolutions with the 30m pixel size used in the study.

Answer: The average monthly precipitation is added together to get the average annual precipitation. The annual average precipitation data were interpolated to a 30 m resolution using geostatistical methods. Due to the small size of the study area and limited spatial variability in precipitation, errors associated with data resolution conversion were not explicitly considered. Lines 139 to 142 are descriptions of Topographic parameters calculated using 30m resolution data, so no relative accuracy or error is involved

While the English usage is mostly understandable, there are minor errors throughout the paper that should be addressed. For example (not a complete list):
Check throughout the paper for the proper use of English articles (a, an, the): _the_ hydrologic cycle, _the_ Random Forest method, etc.

Answer: Has been rechecked and modified
At line 45, please check the units and superscripts on line 45 (10^6 not 106, km^2 or ha?).

Answer：Have been modified

At line 108, what is "soil erosion, soil erosion ditch" (possibly gully formation?).

Answer：Modified as soil erosion and gully

At line 116, rephrase the survey site description in better English.

Answer：Have been modified

At line 149, say Methods not Method and add a short introductory paragraph for this section.

Answer：Have been modified

At line 227, you say that the number of trees has a significant impact, but Table 4 shows that doubling from 50 to 100 trees only reduces the RMSE^oob by <2%.
In Figures 2 and 6, make sure units are given in parenthesis, not after a slash (which can mean "per"): (cm) not /cm, (%) not /%

Answer：Have been modified

Reviewer #2: This manuscript compared several models for predicting the thickness of black soil layer in a small watershed in Northeast China. Authors also explored the soil erosion risk using RUSLE model in the study area. Authors found that the random forest model outperformed the kriging models, with smaller RMSE and larger R². Soil erosion risk assessment revealed that areas with no risk and low risk accounted for 21.91% and 62.21%, respectively. The investigated topic is interesting and important for evaluating and protecting black soil in China. Nevertheless, this work has several major limitations: (1) the work on thickness of black soil layer mapping and soil erosion risk assessment has no linkage. The outcome of thickness map of black soil layer does not provide any useful information on soil erosion risk assessment in the current workflow. However, if you put soil erosion risk assessment as a starting point, then adding this variable as an input for thickness mapping, which is more logical. (2) the current knowledge has been poorly described, limiting the novelty of this work; (3) Please provide both out-of-bag validation and k-fold cross-validation results; (4) there are many mistakes regarding the grammars and duplicated sentences. Please carefully check the writing quality before the resubmission. In summary, this manuscript is not suitable to be published now, but can be reconsidered after re-submission.

Detailed comments:
Title: The use of “Black soil thickness” is confusing. Please consider the use of thickness of black soil layer.

Answer：thickness of black soil layer is more accuracy. Have been modified

Line 29: kriging methods (classical, ordinary, and simple) have been rarely used nowadays.

Answer：With the emergence of new interpolation methods, the use of methods like classical, ordinary and simple Kriging has indeed decreased, but this does not mean that they are no longer valuable. The Kriging method is still an effective tool in the field of geostatistics. Therefore, random forest and Kriging are selected to compare the robustness of their models

Lines 30-31: It is not clear how to connect the work on mapping thickness of black soil layer and soil erosion assessment.

Answer： RUSLE (Revised Universal Soil Loss Equation) model has the characteristics of simple structure, easy parameter acquisition, simple calculation and high precision. It has been widely used in soil erosion modulus estimation at home and abroad. The erosion modulus mainly reflects the influence of erosion factors such as precipitation, slope, and vegetation coverage on soil erosion. These factors drive soil loss by external forcing. Soil thickness, as an intrinsic physical property, plays a pivotal role in determining soil stability, water retention capacity, and erosion resistance. Particularly in regions characterized by intense precipitation or steep topographic gradients, thicker soil profiles demonstrate enhanced resistance to erosional processes, effectively mitigating the impact of external erosive forces. Conventional soil erosion assessment predominantly relies on a unidimensional approach utilizing the soil erosion modulus, which solely quantifies erosion intensity per unit time. However, by integrating soil thickness parameters, a comprehensive two-dimensional evaluation framework of "erosion intensity versus soil layer resistance" can be established, enabling more accurate soil erosion risk assessment. This innovative model classifies erosion risk into four distinct categories: (1) high-risk areas characterized by high erosion modulus coupled with thin soil layers; (2) medium-risk areas exhibiting high erosion modulus with thick soil layers; (3) low-risk areas featuring low erosion modulus and thin soil layers; and (4) risk-free areas demonstrating both low erosion modulus and substantial soil thickness.

Line 32: Please provide the values for RMSE and R2.

Answer：Have been modified

Line 45: 106 km2, 6 should be in superscript. Please use km2 for arable land as well.

Answer：Have been modified

Line 52: It is important to describe the definition of black soil layer. For most readers outside of China, it is not easy to understand the exact meaning.

Answer：Description and definition of the black soil layer have been added.

Lines 57-60: As you stated here, soil erosion plays an important in shaping the thickness of black soil layer, so why not including soil erosion as an input for mapping thickness of black soil layer?

Answer: When the random forest model encounters highly correlated features, it may "overfit" certain variables, leading to a decrease in regression accuracy. The erosion modulus calculated by RUSLE has a strong linear or nonlinear correlation with input variables of the random forest model (such as slope, vegetation index, elevation, precipitation, etc.). Therefore, when it is included in the random forest model, it may lead to information redundancy, which can affect the model's performance. As a result, the RUSLE results were not used as input variables for predicting soil thickness.

Line 61: It would be important to start the paragraph with the fact that soil thickness is the worst predicted soil information as summarized in the review by Chen et al. (2022). Therefore, there is a need to develop new methodology for soil thickness mapping.
Chen, S., Arrouays, D., Mulder, V.L., Poggio, L., Minasny, B., Roudier, P., Libohova, Z., Lagacherie, P., Shi, Z., Hannam, J. and Meersmans, J., 2022. Digital mapping of GlobalSoilMap soil properties at a broad scale: A review. Geoderma, 409, p.115567.

Answer: The research progress of Chen et al. has been added.

Lines 78-81: It is important to acknowledge previous studies on using machine learning model to predict soil thickness. Some relevant references are listed below, and you are explore more other interesting work as well:
Malone, B. and Searle, R., 2020. Improvements to the Australian national soil thickness map using an integrated data mining approach. Geoderma, 377, p.114579.
Liu, F., Zhang, G.L., Song, X., Li, D., Zhao, Y., Yang, J., Wu, H. and Yang, F., 2020. Liu, F., Wu, H., Zhao, Y., Li, D., Yang, J.L., Song, X., Shi, Z., Zhu, A.X. and Zhang, G.L., 2022. Mapping high resolution national soil information grids of China. Science Bulletin, 67(3), pp.328-340.

Answer：Related references on the application of machine learning in the direction of soil thickness in recent years has been added.

Lines 86-90: The current knowledge is missing before stating the objectives.

Answer: Recent research advances and deficiencies related to the research topic have been added in the paragraphs above.

Line 102: N and E are missing after the coordinates.

Answer：Have been modified

Lines 114-123: Please specify the sampling design of the four survey routes.

Answer: The topography of the study area is high in the west and low in the east, high in the south and north and low in the center. Therefore the survey route is in east-west direction.

Line 134: (1) for the equation.

Answer：Have been modified

Lines 137-142: Two sentences are replicated.

Answer：Have been modified

Line 148: In Table 1, why not using the National Soil Information Grids of China at 90 m resolution?

Answer：We apply for the Basic soil property dataset of high-resolution China Soil Information Grids (2010-2018) Data set of Professors Liu Feng and Zhang Ganlin in the National Qinghai-Tibet Plateau Scientific Data Center platform. But the administrator denied our request for 90m resolution data. The data used in this study to calculate the erosion modulus can meet our analytical needs.

Lines 153-154: 500 trees would be more stable.

Answer：We have less data for research, and when the decision tree is 100, the out-of-pocket error has been stabilized, so the number of decision trees is chosen to be 100.

Line 167: In Figure 1, authors indicated the sites for model testing, but here you mentioned that OOB error was used. This is confusing.

Answer：A large number of studies have shown that OOB error is close to cross-validation results. Therefore, the random forest OOB error and Kriging cross validation error are compared as a means of model evaluation.
