# Supplementary material for: Black soil layer thickness prediction and soil erosion risk assessment in a small watershed in Northeast China
Source: PLoS One. 2025 Jun 9;20(6):e0324368. doi: 10.1371/journal.pone.0324368 (PMC12148179; doi:10.1371/journal.pone.0324368)
Supplement: Attachment — Submitted filename: Response_to_Reviewers_auresp_2.docx [file pone.0324368.s003.docx]

(1) the grammar should be greatly improved. Some examples are listed below: “Use the RUSLE erosion equation and soil thickness to create a soil erosion rating scale that integrates external factors and the soil's properties”, “The topography of the study area is high in the west and low in the east, high in the south and north, and low in the center.”.

The grammar of the entire text has been checked and modified.

(2) The reference 10 is not a review paper. Please have a check.

Modified to correct reference.

(3) Lines 104-110: The part is wordy, you can shorten it.

The sentence have been revised to“However, integrating soil thickness parameters establishes a two-dimensional "erosion intensity vs. soil resistance" framework, enabling precise erosion risk classification: (1) high-risk (high erosion modulus, thin soil layers); (2) medium-risk (high modulus, thick layers); (3) low-risk (low modulus, thin layers); and (4) risk-free (low modulus, thick layers).”

(4) Line 88: soil-forming environment should be corrected as soil-forming environmental covariates.

Have been modified.

(5) Line 89: Malong is a typo. It should be corrected as Malone.

Have been modified.

(6) Line 92: quantile random forests should be corrected as QRF since it has been defined in in Line 87.

Have been modified.

(7) Line 131: Fig.1 not Fig 1. Please check similar typos in the manuscript.

Have been modified and checked.

(8) Line 183: There is no need to define RUSLE again.

Have been modified.

(9) Lines 203-215: The problem is that OOB is for random forest, so how you applied for Kriging interpolation. Please make it clear.

OOB is just for random forest. Cross-validation was applied for Kriging interpolation. We also calculate random forest cross-validation to compare with Kriging interpolation.

(10) Table 5: R2 should be corrected as R2.

Have been modified.

(11) You have two c in the figure caption.

Have been modified.

(12) Line 307: Why used the section named “RUSLE result” here? Please check the format throughout the manuscript.

“RUSLE result” have been modified to “Spatial distribution of erosion modulus ”.
